# Supplementary material for: AMPK is a mechano-metabolic sensor linking cell adhesion and mitochondrial dynamics to Myosin-dependent cell migration
Source: Nat Commun. 2023 May 22;14:2740. doi: 10.1038/s41467-023-38292-0 (PMC10202939; doi:10.1038/s41467-023-38292-0)
Supplement: Supplementary file 1 — Supplementary Information [file 41467_2023_38292_MOESM1_ESM.pdf]

SUPPLEMENTAL INFORMATION TITLES AND LEGENDS

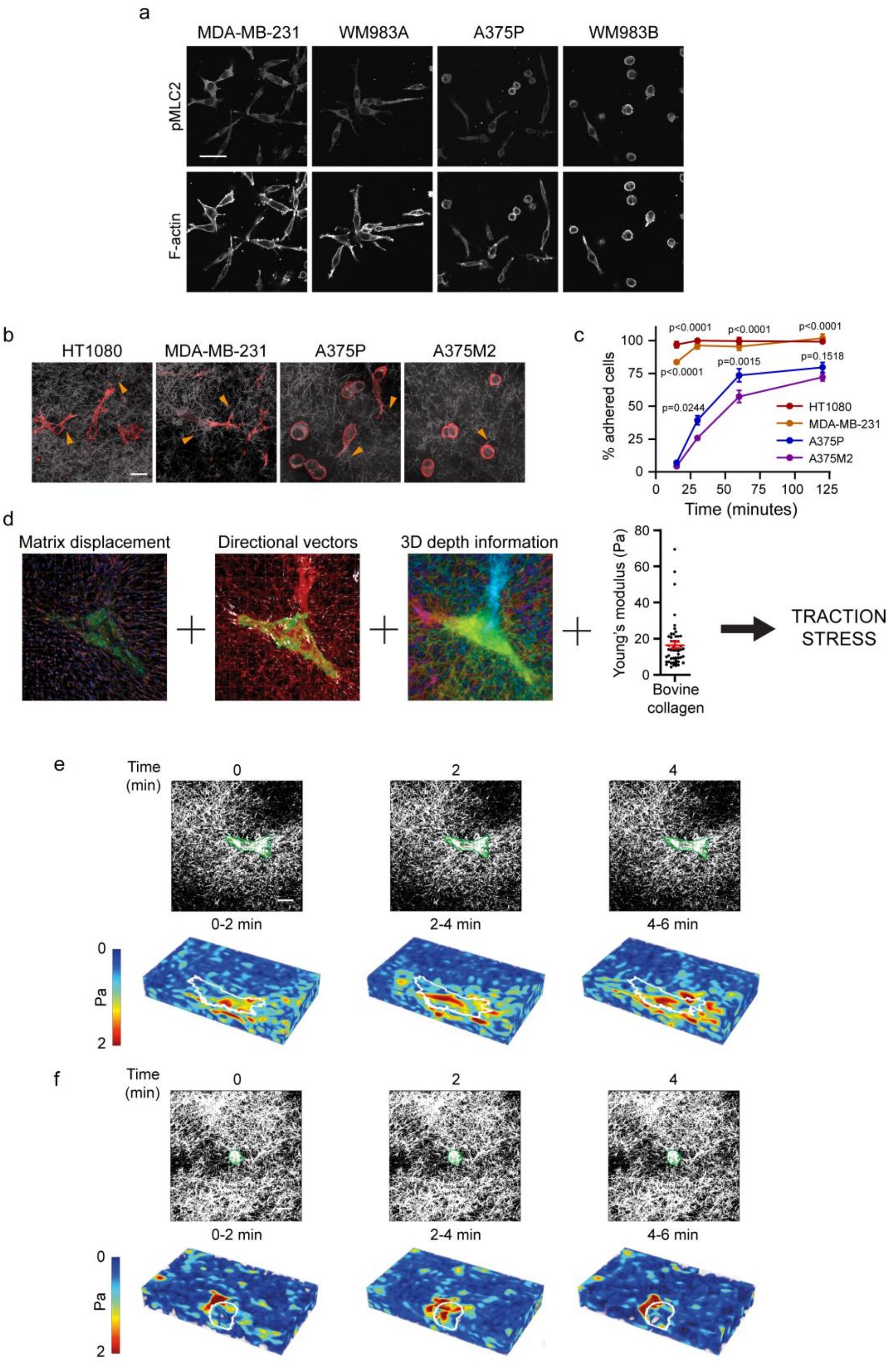

Supplementary Fig. 1: Related to Fig. 1. Cytoskeleton, adhesion and traction stress in 3D migration. Cells seeded on a 3D collagen I matrix. a) pMLC2 and F-actin confocal images in

a panel of cell lines (n=3). Scale bar=50μm. b) Representative images of collagen fibres (grey) and F-actin (red) for the quantification of points of attachment between cells and matrix (n=3). Arrows indicate representative points of attachment. Scale bar=20μm. c) Percentage of adhered cells at indicated times after seeding cells on a matrix of collagen I (n=3). d) Representative scheme showing incorporation of parameters to calculate traction stress. Dot plot represents matrix stiffness of bovine collagen I measured using atomic force microscopy (AFM) (51 measurements at random positions from n=4 independent collagen gels). e-f) 3D reconstructions of traction stress allow for visualisation of distinct stress patterns associated with different modes of migration. Representative maximum intensity projections showing individual collagen I fibres surrounding HT1080 (e top) and A375M2 (f top) cells at the indicated time points. Cell profile highlighted in green. Scale bar=20μm. Representative 3D traction stress magnitude maps reconstructed from a z-stack corresponding to the mid-volume of HT1080 (e bottom) and A375M2 (f bottom) cells migrating through collagen I, between time points of 0-6 minutes. Colour bar represents traction stress magnitude (Pascal, Pa). Graph (c) and dot plot (d) show mean±SEM. p values by two-way ANOVA with Dunnett's correction versus A375M2 (c). All n are indicative of independent experiments unless otherwise stated.

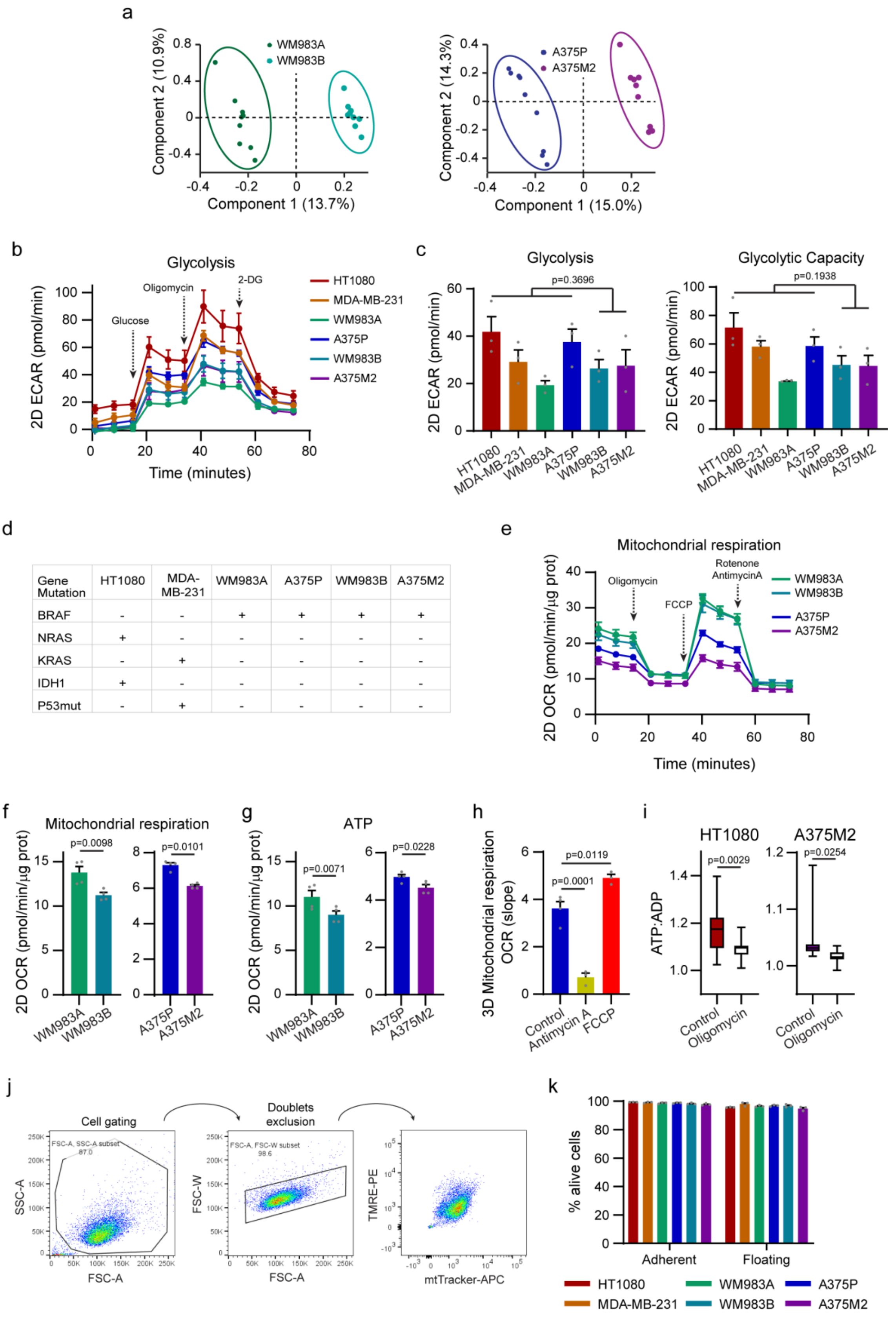

Gene Mutation	HT1080	MDA-MB-231	WM983A	A375P	WM983B	A375M2
BRAF	-	-	+	+	+	+
NRAS	+	-	-	-	-	-
KRAS	-	+	-	-	-	-
IDH1	+	-	-	-	-	-
P53mut	-	+	-	-	-	-

Supplementary Fig. 2: Related to Fig. 2. Different modes of migration and mitochondrial metabolism. a) Score scatter plot showing PLS-DA analysis of the full spectra of ^{1}H-NMR

data, where peak intensities of metabolites represent the variables. Spatial distribution of the samples is based on the metabolite pattern (n=3 with 3 technical replicates). b) Extracellular acidification rate (ECAR) profile in a panel of cell lines using a Seahorse analyser (n=3). c) Glycolytic rate (left) and glycolytic capacity (right) in a panel of cell lines using a Seahorse analyser (n=3). d) Table showing the mutational status of the cell lines used in this study. e) Oxygen consumption rate (OCR) profile for the indicated cell lines using a Seahorse analyser (n=4). f-g) OCR (f) and ATP production from mitochondrial respiration (g) in the indicated cell lines using a Seahorse analyser, normalised by protein content (n=4). h) OCR from A375P cells embedded in a 3D collagen I matrix treated with Antimycin A (1μM) or FCCP (0.75μM) (n=3). i) Quantification of ATP:ADP ratio in HT1080 and A375M2 cells expressing Perceval HR biosensor embedded in a 3D collagen I matrix treated with Oligomycin (1μM) (18, 13, 18, 14 cells pooled from n=3). j) Gating strategy used in Fig. 2h. k) Percentage of alive cells after 24 hours under adherent or floating conditions analysed by FACS (n=3). Graphs (b,c,e,f,g,h,k) show mean±SEM. Box plots (i) show median (centre line), interquartile range (box) and min-max values (whiskers). p values were calculated using two-tailed tests (c,f-g,i). p value by unpaired t-test comparing elongated-mesenchymal versus rounded-amoeboid cells (c), paired t-test (f,g), unpaired t-test (i) and one-way ANOVA with Dunnett’s correction (h). All n are indicative of independent experiments unless otherwise stated.

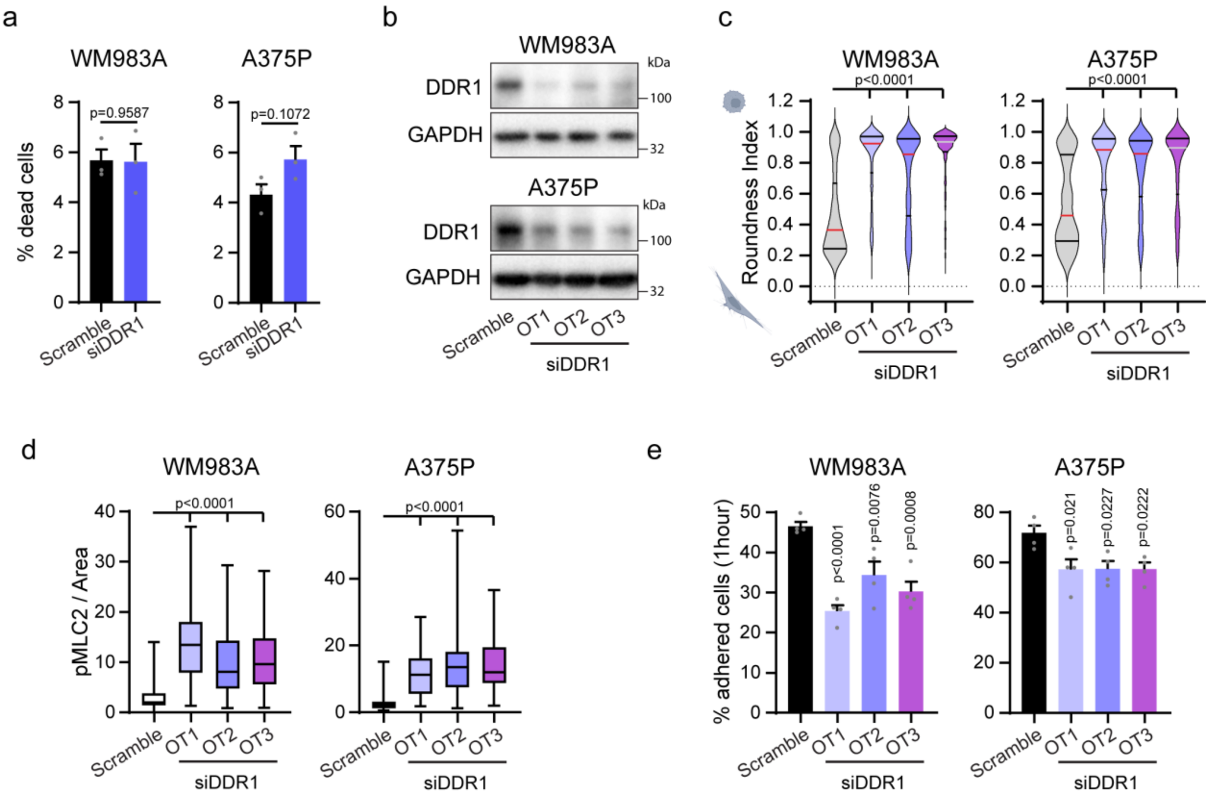

Supplementary Fig. 3: Related to Fig. 3. DDR1 controls adhesion levels and energy demands. a) After DDR1 knock-down, percentage of cell death analysed by FACS (n=3). b) DDR1 protein levels after DDR1 knock-down (n=3). c) Quantification of cell morphology (331, 285, 291, 314 cells (WM983A) and 201, 254, 258, 238 cells (A375P) pooled from n=3). d) Quantification of pMLC2 immunofluorescence signal normalized by cell area (102, 110, 87, 69 cells (WM983A) and 93, 93, 82, 58 cells (A375P) pooled from n=3). e) Quantification of adhered cells after 1 hour seeding on a collagen I matrix (n=4). Graphs (a,e) show mean±SEM. Violin plots (c) show median with interquartile range. Box plots (d) show median (centre line), interquartile range (box) and min-max values (whiskers). p value by two-tailed unpaired t-test (a), Kruskal-Wallis with Dunn's multiple comparisons test (c,d) and one-way ANOVA with Dunnett's correction (e). All n are indicative of independent experiments unless otherwise stated.

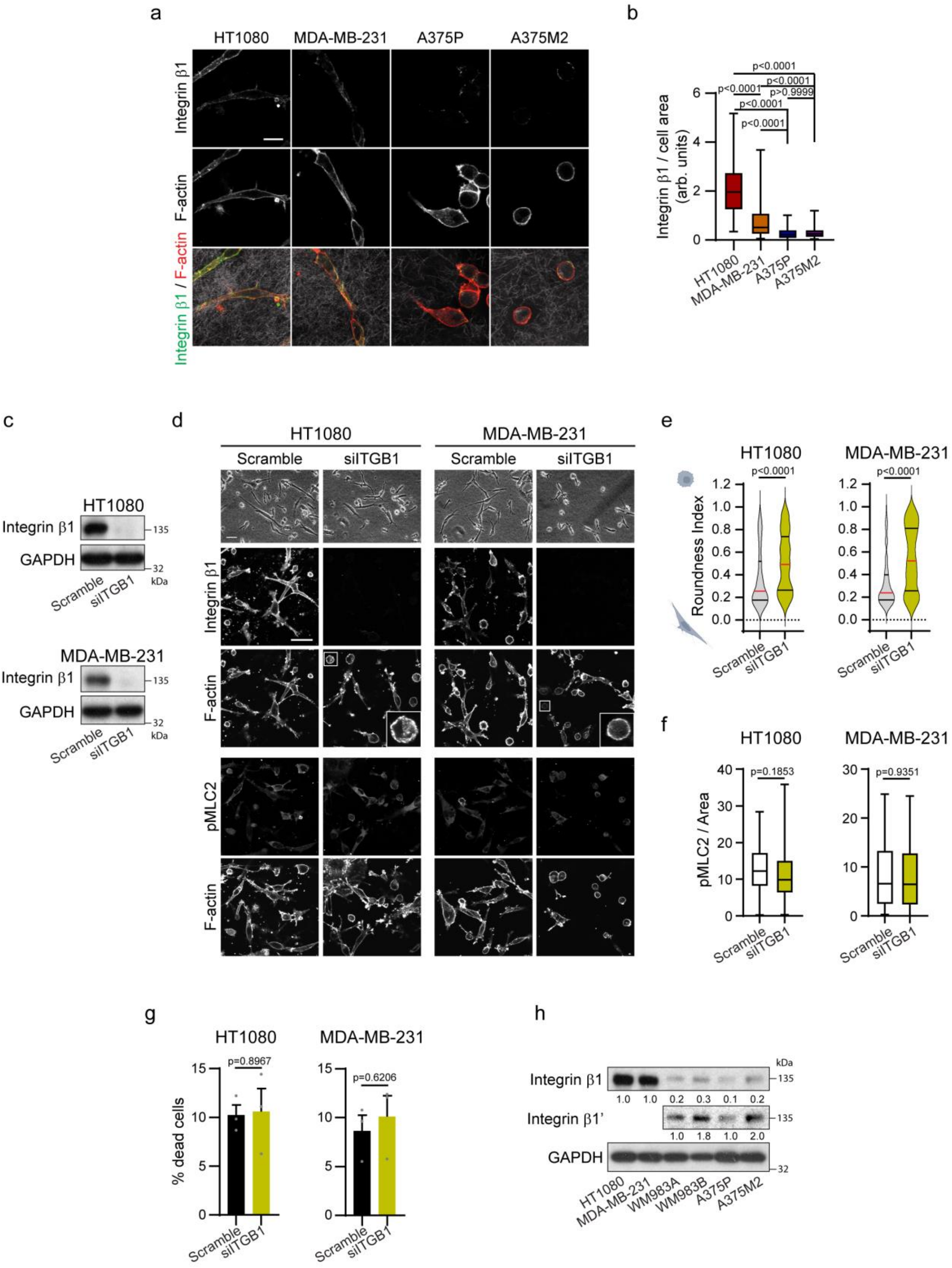

Supplementary Fig. 4: Related to Fig. 3. Loss of integrin β1 increases cell rounding without impacting on cortical Myosin II activity. Cells seeded on a 3D collagen I matrix. a) Representative images of integrin β1 (green), F-actin (red) and collagen fibres (grey) (n=3).

Scale bar=20μm. b) Quantification of integrin β1 normalized by cell area by immunofluorescence (74, 90, 160, 105 cells pooled from n=3). c) Integrin β1 protein levels after integrin β1 knock-down in HT1080 and MDA-MB-231 cells (n=3). d) After ITGB1 knock-down, representative bright field images (d top), integrin β1, pMLC2 and F-actin immunofluorescence images (d bottom) (n=3). Scale bar = 50 μm. Inset showing F-actin in a non-blebbing rounded cell. e-g) After *ITGB1* knock-down, quantification of cell morphology (181, 161 cells (HT1080) and 167, 161 cells (MDA-MB-231) pooled from n=3) (e), quantification of pMLC2 immunofluorescence signal normalized by cell area (50, 50, 55, 54 cells pooled from n=3) (f) and percentage of cell death analysed by FACS (n=3) (g). h) Integrin β1 protein levels (n=5). Quantification normalized by GAPDH. Box plots (b,f) show median (centre line), interquartile range (box) and min-max values (whiskers). Violin plots (e) show median with interquartile range. Graphs (g) show mean±SEM. p values were calculated using two-tailed tests (g-f). p value by unpaired t-test (g), Mann-Whitney test (e,f), Kruskal-Wallis with Dunn's multiple comparisons test (b). All n are indicative of independent experiments unless otherwise stated.

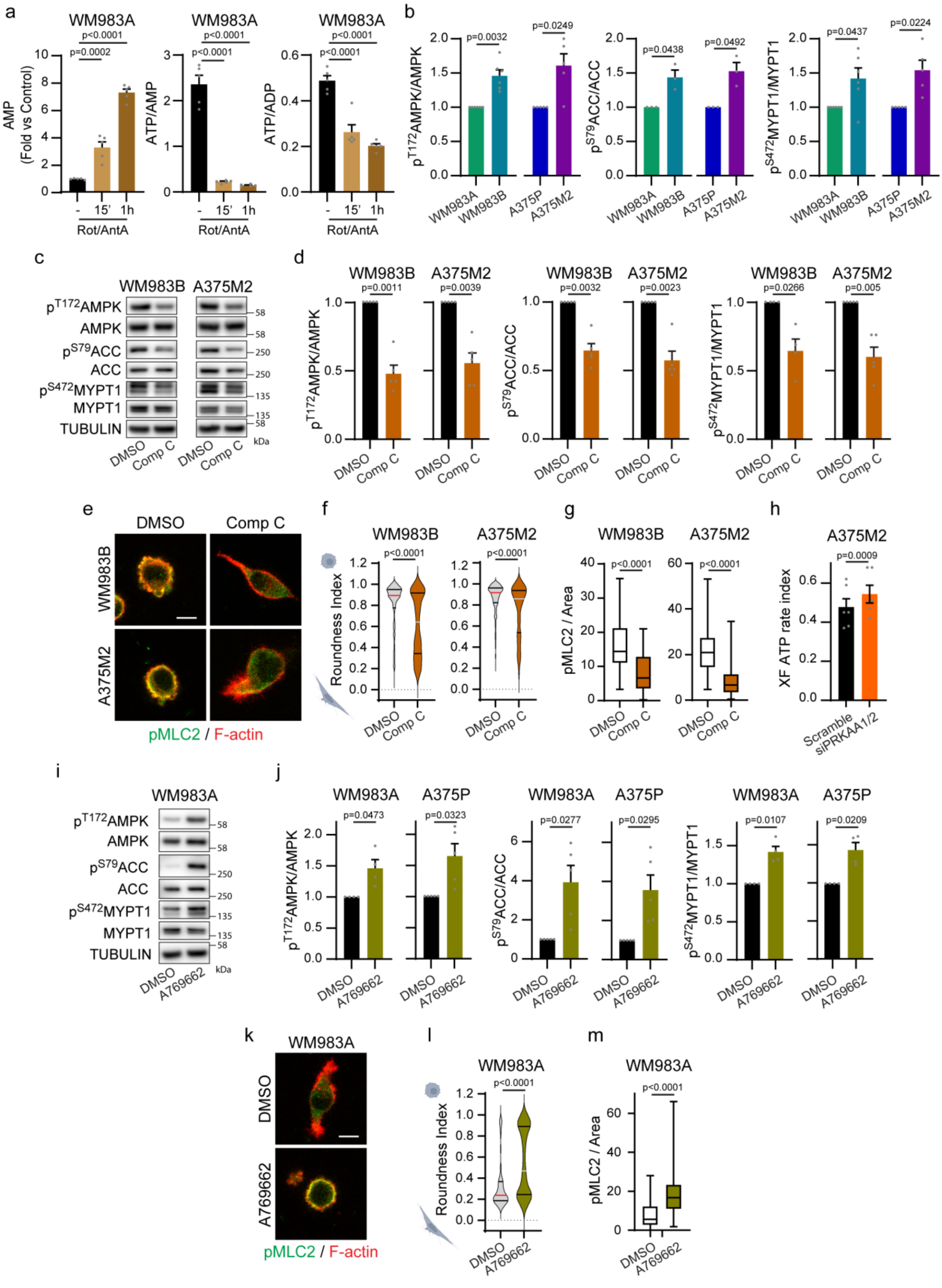

Supplementary Fig. 5: Related to Fig. 4. ATP levels control plasticity of cell migration through AMPK. a) AMP, ATP/AMP and ATP/ADP in WM983A cells treated with Rotenone

(0.5μM) and Antimycin A (0.5μM) for the indicated time, analysed by LC-MS (5 replicates/condition). b) Western blot quantification of the indicated proteins in rounded-amoeboid versus elongated-mesenchymal cells (WM983 n=6, A375 n=5). c-d) Western blot (c) and quantification (d) of the indicated proteins in WM983B and A375M2 cells upon AMPK inhibition (Compound C (Comp C) 2μM, 24hours) (WM983B n=5, A375M2 n=5). e) Cells seeded on a collagen I matrix. Immunofluorescence images showing pMLC2 (green) and F-actin (red) after Comp C treatment (2μM, 24hours) (n=3). Scale bar=10μm. f-g) After Comp C treatment, quantification of cell morphology (379, 306 cells (WM983B) and 308, 193 cells (A375M2) pooled from n=3) (f) and quantification of pMLC2 immunofluorescence signal normalized by cell area (91, 83 cells (WM983B) and 77, 78 cells (A375M2) pooled from n=3) (g). h) XF ATP rate index, indicative of the ratio between mitochondrial and glycolytic ATP, upon AMPK knock-down in A375M2 cells (n=6). i-j) Western blot (i) and quantification (j) of the indicated proteins upon AMPK activation (A769662 10μM, 30 minutes) (n=4). k) Cells grown on a collagen I matrix. Immunofluorescence images showing pMLC2 (green) and F-actin (red) after A769662 treatment (10μM, 24hours) (n=3). Scale bar=10μm. l-m) After A769662 treatment, quantification of cell morphology (167, 233 cells pooled from n=3) (l) and quantification of pMLC2 immunofluorescence signal normalized by cell area (81, 84 cells pooled from n=3) (m). Graphs (a,b,d,h,j) show mean±SEM. Violin plots (f,l) show median with interquartile range. Box plots (g,m) show median (centre line), interquartile range (box) and min-max values (whiskers). p values were calculated using two-tailed tests (b,d,f-h,j,l-m). p value by one sample t-test (b,d,j), paired t-test (h) and Mann-Whitney test (f,g,l,m) and one-way ANOVA with Dunnett's correction (a). All n are indicative of independent experiments unless otherwise stated.

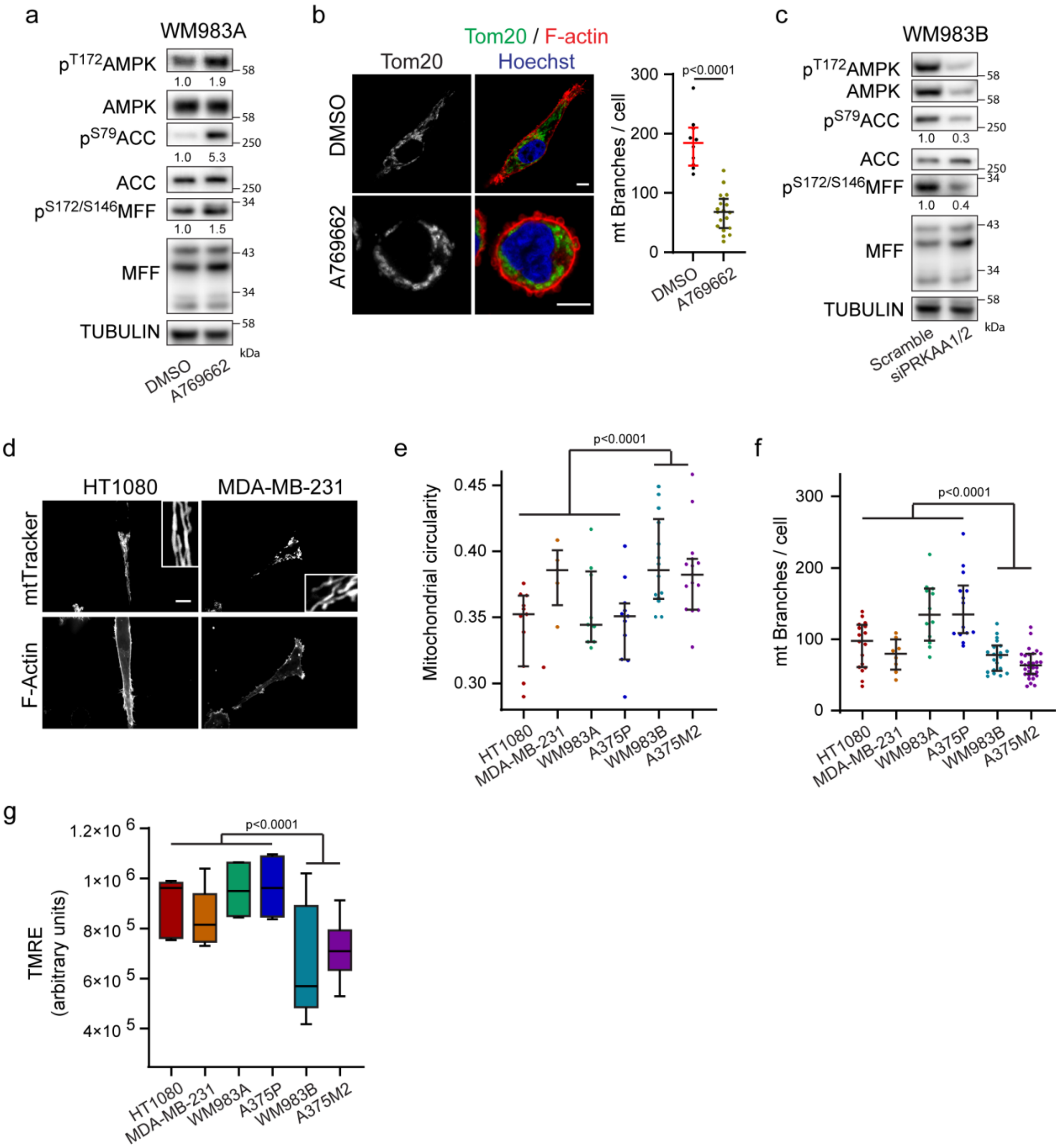

Supplementary Fig. 6: Related to Fig. 5. Mitochondrial dynamics in 3D. a) Upon AMPK activation (A769662 10µM, 30minutes) in WM983A cells, western blot showing levels of the indicated proteins (n=3). b) Cells seeded on a collagen I matrix. (Left) Representative images of mitochondrial network (Tom20, green), F-actin (red) and nucleus (Hoechst, blue) after A769662 treatment (10µM, 24hours). Scale bar=5µm. (Right) Quantification of mitochondrial branches per cell from Tom20 staining (10, 19 cells pooled from n=3). c) Western blot of the indicated proteins after AMPK knock-down (si*PRKAA1/2*) in WM983B cells (n=3). d) Live

cell imaging of mitochondria using MitoTracker Deep Red of the indicated cell lines stably transfected with LifeAct-GFP and seeded on a collagen I matrix (n=3). Scale bar=10μm. e) Quantification of mitochondrial circularity (11, 5, 9, 14, 11, 15 cells pooled from n=3). f) Quantification of mitochondrial branches per cell from Tom20 staining (11, 8, 12, 20, 14, 30 cells pooled from n=3). g) Quantification of TMRE fluorescence intensity by immunofluorescence, as readout of mitochondrial activity (n=3). Western blots quantification normalized by the corresponding total protein (a,c). Dot plots (b,e,f) show median with interquartile range (each dot represents a single cell). Box plots (g) show median (centre line), interquartile range (box) and min-max values (whiskers). p value by two-tailed unpaired t-test (b,e,f,g). All n are indicative of independent experiments unless otherwise stated.

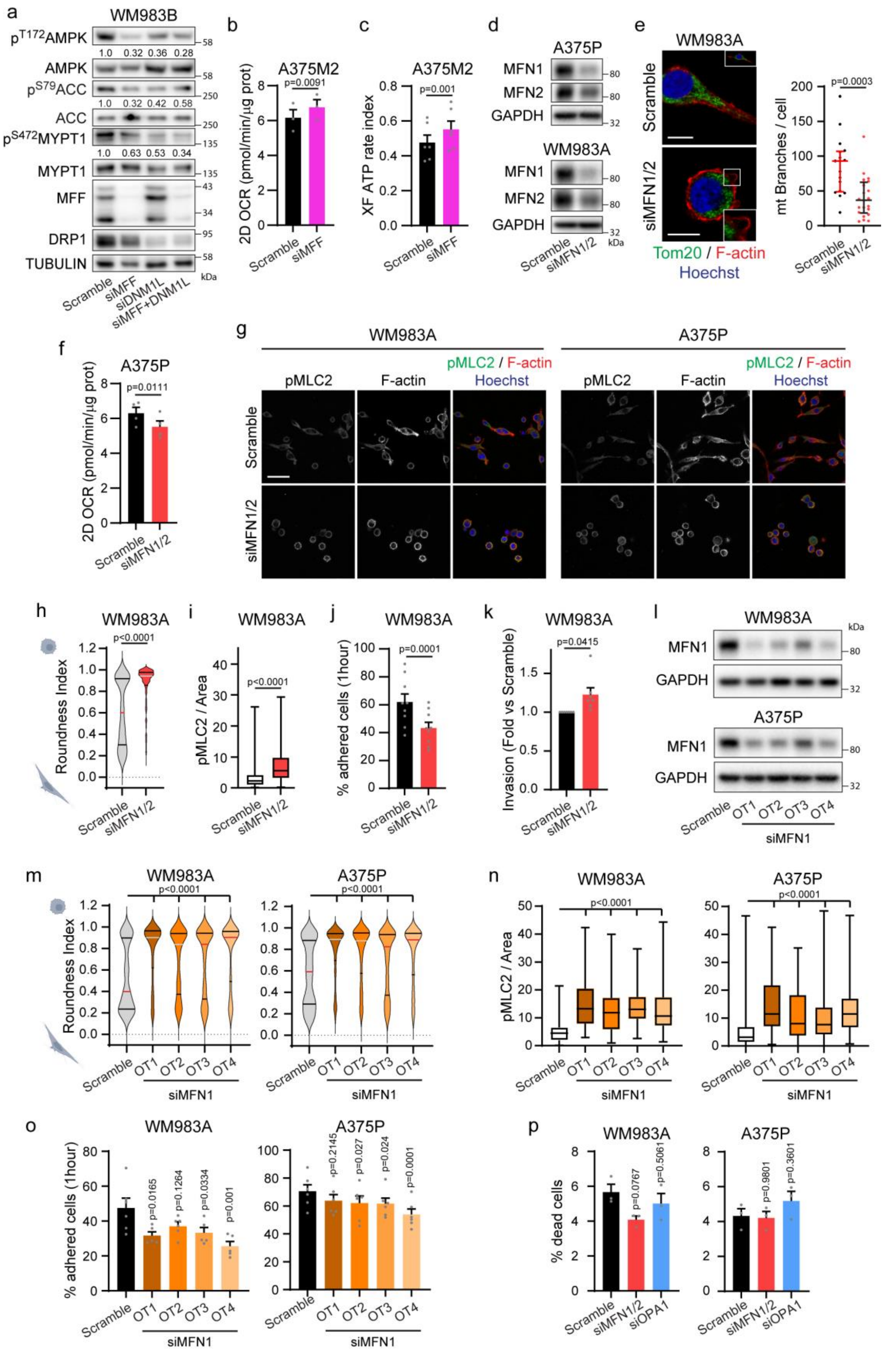

Supplementary Fig. 7: Related to Fig. 6 and 7. Mitochondrial dynamics control the cytoskeleton and invasion. a) Western blot after the indicated knock-downs (n=3).

Quantifications normalized versus total protein. b-c) Oxygen consumption rate (OCR) (n=3) (b) and XF ATP rate index (c) after MFF knock-down (n=6). d) Mitochondrial fusion protein levels after MFN1/2 knock-down (n=4). e) (Left) Mitochondrial network (Tom20, green), F-actin (red) and nucleus (Hoechst, blue) after MFN1/2 knock-down. Inset shows whole cell (Scramble) and bleb protrusion (siMFN1/2). Scale bars=10μm. (Right) Quantification of mitochondrial branches per cell from Tom20 staining (17, 22 cells pooled from n=3). f) OCR after MFN1/2 knock-down (n=4). g) Cells seeded on a collagen I matrix. Immunofluorescence of pMLC2 (green), F-actin (red) and Hoechst (blue) after MFN1/2 knock-down (n=3). Scale bar=50μm. h-k) After MFN1/2 knock-down, quantification of cell morphology (342, 313 cells pooled from n=3) (h), pMLC2 immunofluorescence signal normalized by cell area (103, 102 cells pooled from n=3) (i), adhered cells after 1 hour seeding on a collagen I matrix (n=9) (j) and 3D invasion index (n=7) (k). l) MFN1 protein levels after using four On-Target siMFN1 (n=3). m-o) After MFN1 knock-down, quantification of cell morphology (260, 229, 234, 218, 224 cells (WM983A) and 405, 357, 311, 325, 358 cells (A375P) pooled from n=3) (m), pMLC2 by immunofluorescence (104, 106, 112, 107, 113 cells (WM983A) and 132, 134, 124, 138, 121 cells (A375P) pooled from n=3) (n) and adhered cells after 1 hour seeding on a collagen I matrix (n=5 (WM983A) and n=6 (A375P)) (o). p) Percentage of cell death after the indicated knock-downs (n=3). Graphs (b,c,f,j,k,o,p) show mean±SEM. Dot plots (e) show median with interquartile range (each dot represents a single cell). Violin plots (h,m) show median with interquartile range. Box plots (i,n) show median (centre line), interquartile range (box) and min-max values (whiskers). p values were calculated using two-tailed tests (b,c,e,f,h-k). p value by one sample t-test (k), paired t-test (b,c,f,j), unpaired t-test (e), Mann Whitney test (h,i), Kruskal-Wallis with Dunn’s multiple comparisons test (m,n) and one-way ANOVA with Dunnett’s correction (o,p). All n are indicative of independent experiments unless otherwise stated.

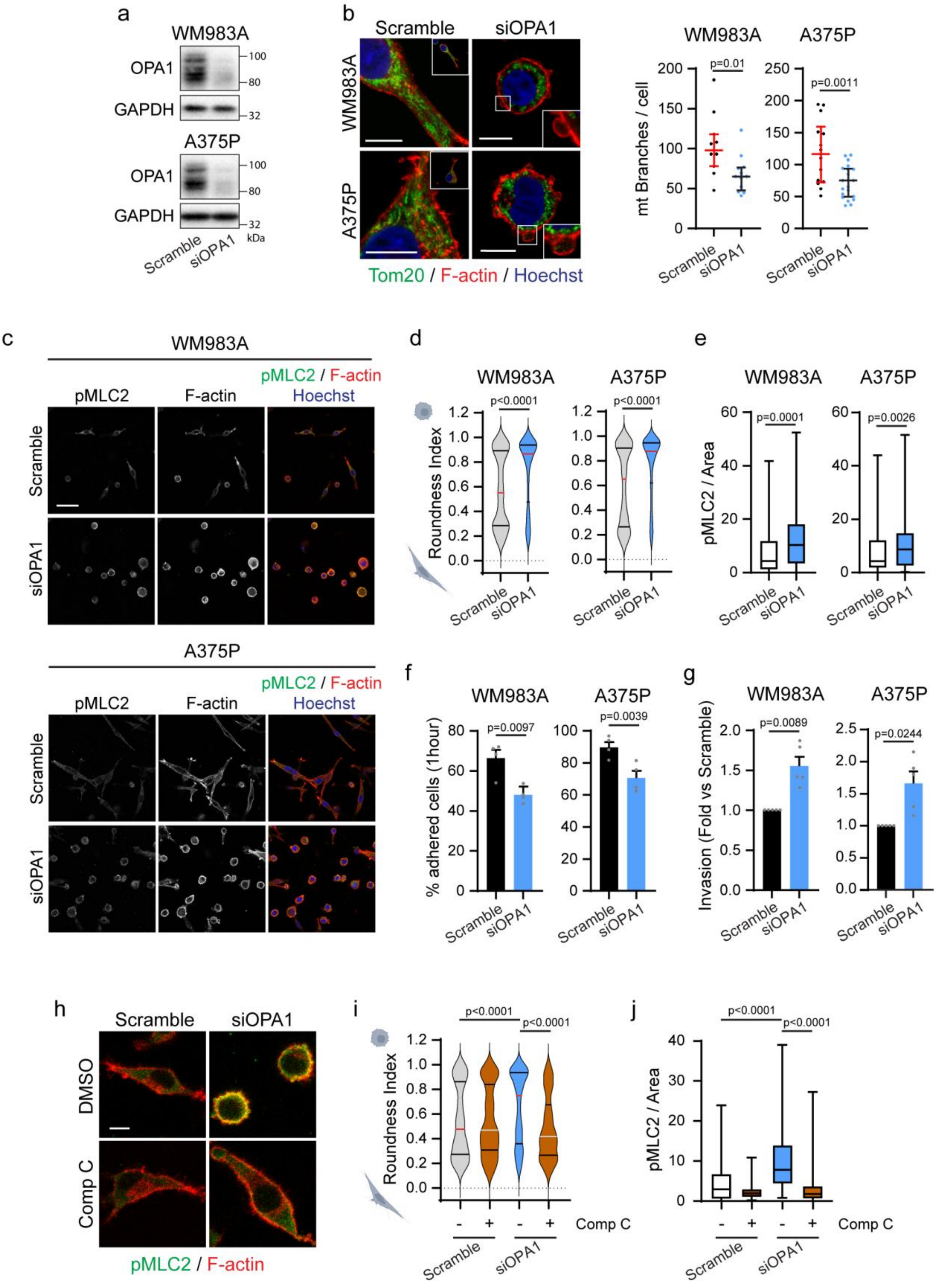

Supplementary Fig. 8: Related to Fig. 7. Perturbing OPA1 expression alters invasion in 3D. a) OPA1 protein levels after OPA1 knock-down in WM983A and A375P cells (n=4). Cells

seeded on a collagen I matrix. b) (Left) Representative images of mitochondrial network (Tom20, green), F-actin (red) and nucleus (Hoechst, blue) after OPA1 knock-down. Inset shows whole cell for Scramble and bleb protrusion for siOPA1. Scale bars=10μm. (Right) Quantification of mitochondrial branches per cell from Tom20 staining (11, 12, 14, 21 cells pooled from n=3). c) Immunofluorescence images showing pMLC2 levels (green), F-actin (red) and Hoechst (blue) after OPA1 knock-down (n=3). Scale bar=50μm. d-g) After OPA1 knock-down in WM983A and A375P cells, quantification of cell morphology (373, 387 cells (WM983A) and 457, 432 cells (A375P) pooled from n=3) (d), quantification of pMLC2 immunofluorescence signal normalized by cell area (107, 108 cells (WM983A) and 157, 145 cells (A375P) pooled from n=3) (e), quantification of adhered cells after 1 hour seeding on a collagen I matrix (n=4) (f) and 3D invasion index into a collagen I matrix (n=5) (g). h) Cells seeded on a collagen I matrix. Immunofluorescence images showing pMLC2 (green) and F-actin (red) after OPA1 knock-down and Comp C treatment (2μM, 24hours) (n=3). Scale bar=10μm. i-j) After OPA1 knock-down and Comp C treatment, quantification of cell morphology (299, 222, 277, 275 cells pooled from n=3) (i) and quantification of pMLC2 immunofluorescence signal normalized by cell area (82, 75, 84, 76 cells pooled from n=3) (j). Dot plots (b) show median with interquartile range (each dot represents a single cell). Violin plots (d,i) show median with interquartile range. Box plots (e,j) show median (centre line), interquartile range (box) and min-max values (whiskers). Graphs (f,g) show mean±SEM. p values were calculated using two-tailed tests (b,d-g). p value by one sample t-test (g), unpaired t-test (b), paired t-test (f), Mann-Whitney test (d,e) and Kruskal-Wallis with Dunn's multiple comparisons test (i,j). All n are indicative of independent experiments unless otherwise stated.

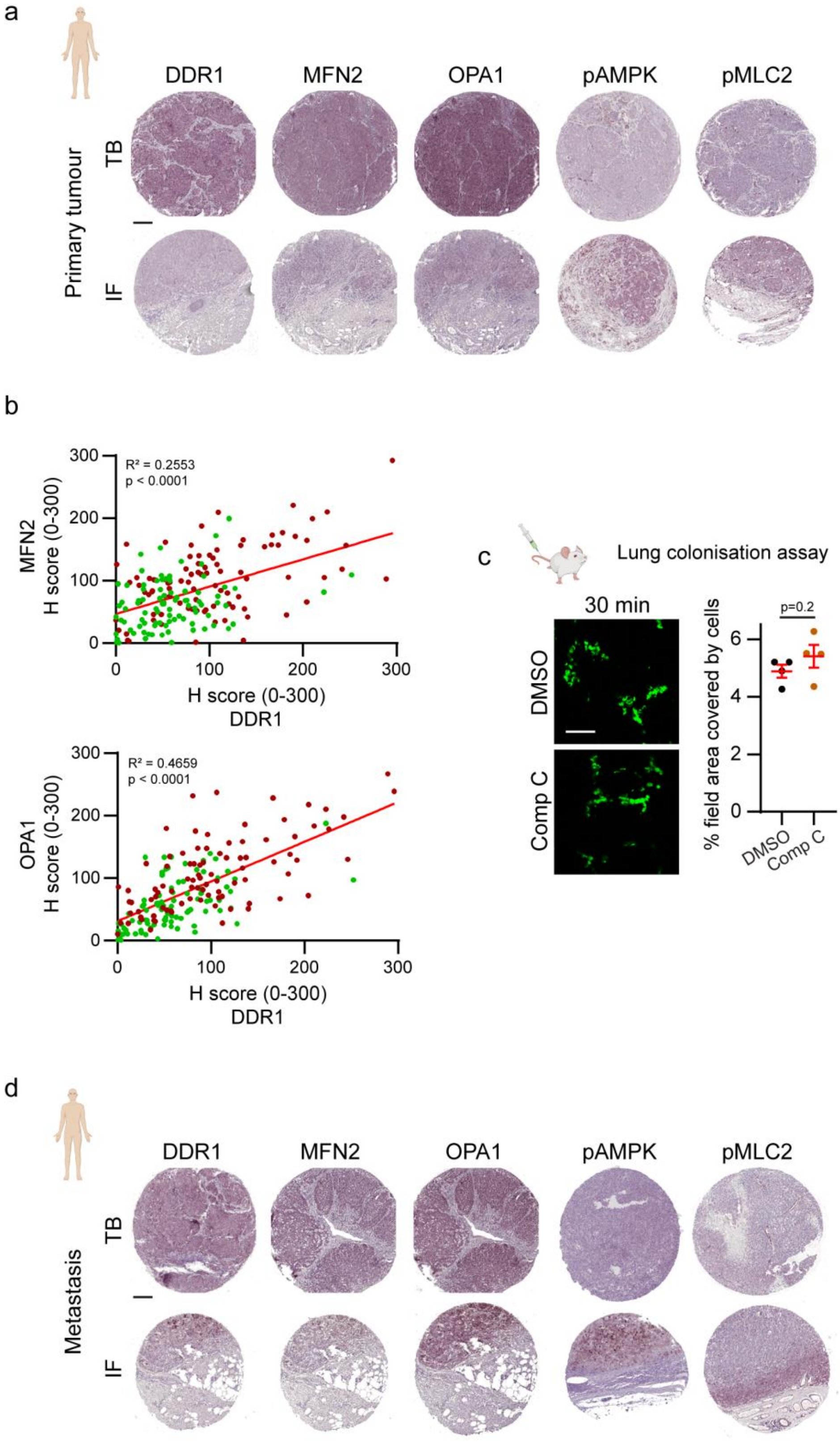

Supplementary Fig. 9: Related to Fig. 8 and 9. Adhesion, AMPK and mitochondrial dynamics *in vivo* and in melanoma. a) Representative images of DDR1, MFN2, OPA1, pAMPK, and pMLC2 expression in matched tumour body (TB) and invasive front (IF) of

primary tumours from human melanoma tissue microarray. Scale bar=200µm. b) Scatter plot representing correlation between MFN2 and DDR1 (top) and between OPA1 and DDR1 (bottom) H score from the human tissue microarray. Brown dots indicative of H score in the TB and green dots indicative of H score in the IF. Pearson correlation was used to calculate R^2 and p value. c) (Left) Representative confocal images of mouse lungs 30 minutes after tail vein injection of 5-chloromethylfluorescein diacetate (CMFDA)-Green labelled A375M2 pre-treated with Comp C (2µM, 24hours) and (right) percentage of field area covered by cells (10 fields/mouse/condition, 2 mice/condition, n=2 independent experiments). Scale bar=100µm. d) Representative images of DDR1, MFN2, OPA1, pAMPK, and pMLC2 expression in matched TB and IF of metastasis from the human melanoma tissue microarray. Scale bar=200µm. Dot plot (c) shows mean±SEM (each dot represents a single mouse). p values were calculated using two-tailed tests (b-c). p value by Mann-Whitney test (c).

SUPPLEMENTARY TABLES WITH TITLE AND LEGEND

Supplementary Table 1. Related to Methods. Clinical information for primary melanoma patients.

**Age** (years)	mean ± SD	63.7 ± 17.9
**Gender**	Male	26 (56.5%)
	Female	20 (43.5%)
**Location**	Trunk	22 (47.8%)
	Head and neck	13 (28.3%)
	Lower limb	4 (8.7%)
	Upper limb	4 (8.7%)
	Foot	2 (4.3%)
	Hand	1 (2.2%)

Supplementary Table 2. Related to Methods. Clinical information for metastatic melanoma patients.

**Age** (years)	mean ± SD	63.1 ± 16.3
**Gender**	Male	25 (55.6%)
	Female	20 (44.4%)
**Location**	Lymph node	39 (86.7%)
	Cutaneous/subcutaneous	5 (11.1%)
	Lung	1 (2.2%)

Supplementary Table 3. Related to Methods. List of siRNA sequences.

Target	siRNA sequence
siGENOME non-targeting Control (Scramble)	5'-UGGUUUACAUGUCGACUAA-3'
PRKAA1	siGENOME SMARTpool:<br>5'-CAAAGUCGACCAAAUGAUA-3'<br>5'-GUAGAGCAAUCAAACAAUU-3'<br>5'-GACAAGCACUUACUCCAAA-3'<br>5'-ACAAUUGGAUUAUGAAUGG-3'
PRKAA2	siGENOME SMARTpool:<br>5'GUACCUACGUUAUUUAAGA-3'<br>5'-GGAAGGUAGUGAAUGCAUA-3'<br>5'-GACAGAAGAUUCGCAGUUU-3'<br>5'-ACAGAAGAUUCGCAGUUUA-3'
DNM1L	siGENOME SMARTpool:<br>5'-GAAAGAAGCAGCUGAUAUG-3'<br>5'-GGAGCCAGCUAGAUAUUAA-3'<br>5'-CAAAGGCAGUAAUGCAUUU-3'<br>5'-CGUAAAAGGUUGCCUGUUA-3'
MFF	siGENOME SMARTpool:<br>5'-CGACAGAUAAUCAAACUAA-3'<br>5'-UCCAAAUGCUAGUGUGAUA-3'<br>5'-CGUCAGGUAUGGCAUUUCA-3'<br>5'-AGUCGAAUUCAGUACGAAA-3'
MFN1	siGENOME SMARTpool:<br>5'-GAAGAGCUCUGUUAUCAAU-3'<br>5'-GCACAGAUGUCACUACAGA-3'<br>5'-GAUACUAGCUACUGUGAAA-3'<br>5'-CUGGAUAGCUGGAUUGAUA-3'
MFN2	siGENOME SMARTpool:<br>5'-ACUAUAAGCUGCGAAUUAA-3'<br>5'-GAUCAGGCGCCUCUCUGUA-3'<br>5'-GGUUACCUAUCCAAAGUGA-3'<br>5'-CAACUAUGACCUAAACUGU-3'
OPA1	siGENOME SMARTpool:<br>5'-GAACAGCUCUGAAAGCAUU-3'<br>5'-GAAACUGAAUGGAAGAAUA-3'<br>5'-AAAGAAGGCUGUACCGUUA-3'<br>5'-AAACACAGCUCUGGAAUAA-3'
DDR1	siGENOME SMARTpool:<br>5'-UGAAAGAGGUGAAGAUCAU-3'<br>5'-GGGACACCCUUUGCUGGUA-3'<br>5'-GAAUGUCGCUUCCGGCGUG-3'<br>5'-UGGUUACUCUUCAGCGAAA-3'
Integrin β1 (ITGB1)	siGENOME SMARTpool:<br>5'-GAACAGAUCUGAUGAAUGA-3'<br>5'-CAAGAGAGCUGAAGACUAU-3'<br>5'-GAAGGGAGUUUGCUAAAUU-3'<br>5'-CCACAGACAUUUACAUUAA-3'
ON-TARGET (OT) non-targeting Control (Scramble)	5'-UGGUUUACAUGUCGACUAA-3'
MFN1	ON-TARGETplus individual sequences:<br>OT1: 5'-CGAUGAAGUAAACGCCUUA-3'<br>OT2: 5'-CAUGAUAGGAGGAAACGAA-3'<br>OT3: 5'-CAGAAUAUAUGGAAGACGU-3'<br>OT4: 5'-GGAAGUUCUUAGUGCUAGA-3'
DDR1	On-Target individual sequences:<br>OT1: 5'-GGGACACCCUUUGCUGGUA-3'<br>OT2: 5'-GAAUGUCGCUUCCGGCGUC-3'<br>OT3: 5'-AAGAGGAGCUGACGGUUCA-3'
