# Supplementary material for: AMPK is a mechano-metabolic sensor linking cell adhesion and mitochondrial dynamics to Myosin-dependent cell migration
Source: Nat Commun. 2023 May 22;14:2740. doi: 10.1038/s41467-023-38292-0 (PMC10202939; doi:10.1038/s41467-023-38292-0)
Supplement: Supplementary file 8 — Description of Additional Supplementary files [file 41467_2023_38292_MOESM8_ESM.pdf]

Description Of Additional Supplementary files

Fie Name: Supplementary Movie 1.
Description: Collagen I matrix displacement associated with elongatedmesenchymal movement. 3D time-lapse video of HT1080 cell expressing LifeAct-GFP (F-actin, green) embedded within 3D Collagen I matrix. In grey, collagen I fibres imaged by collecting the reflected/backscatter light from the matrix using laser-scanning confocal microscopy.

Fie Name: Supplementary Movie 2.
Description: Stress vectors associated to matrix displacement from video 1.

Fie Name: Supplementary Movie 3.
Description: Collagen I matrix from video 1 with colour coded depth information.

Fie Name: Supplementary Movie 4.
Description: Elongatedmesenchymal cell showing elongated mitochondria forming a network. A 375P cell expressing LifeAct-GFP (F-actin, green) stained with Mitotracker Deep Red (red) and H oechst (blue). First half of the video shows 3D reconstruction of elongated-mesenchymal (Mitotracker Deep Red) and nucleus (Hoechst). Second half of the video showing actin structures (LifeAct-GFP expression), in addition to mitochondria and nucleus.

Fie Name: Supplementary Movie 5.
Description: Rounded-amoeboid cell showing motile punctate fragmented mitochondria. A375M2 cell expressing LifeAct-GFP (F-actin, green) stained with Mitotracker Deep Red (red) and Hoechst (blue). First half of the video shows 3D reconstruction of highly motile fragmented mitochondria (Mitotracker Deep Red) and nucleus (Hoechst). Second half of the video showing bleb actin structures (LifeAct-GFP expression). In addition to mitochondria and nucleus.
